# Structural insights into cholesterol transport and hydrolase activity of a putative human RNA transport protein SIDT1

**DOI:** 10.1038/s41421-024-00647-2

**Published:** 2024-02-20

**Authors:** Wenxia Liu, Mengyuan Tang, Jiening Wang, Fangfang Wang, Gaojie Song, Xiaokang Zhang, Shan Wu, Heng Ru

**Affiliations:** 1 Zhejiang Provincial Key Laboratory for Cancer Molecular Cell Biology, Life Sciences Institute, Zhejiang University, Hangzhou, Zhejiang China; 2 State Key Laboratory of Biocatalysis and Enzyme Engineering, Hubei Collaborative Innovation Center for Green Transformation of Bio-Resources, Hubei Key Laboratory of Industrial Biotechnology, School of Life Sciences, Hubei University, Wuhan, Hubei China; 3 Shanghai Key Laboratory of Regulatory Biology, Institute of Biomedical Sciences and School of Life Sciences, East China Normal University, Shanghai, China; 4 Interdisciplinary Center for Brain Information, The Brain Cognition and Brain Disease Institute, Shenzhen Institute of Advanced Technology, Chinese Academy of Sciences, Shenzhen, Guangdong China

**Keywords:** Cryoelectron microscopy, Transport carrier

Dear Editor,

Sphingolipids and cholesterols are essential components of cell membranes. The amounts and distributions of both kinds of lipids within the membrane can be regulated by lipid hydrolases and cholesterol transport proteins, which in turn affect the dynamics and morphology of the cell membrane^1^. Consequently, they are pivotal in facilitating crucial cellular processes such as endocytosis, exosome formation, and transport^2^. The mammalian integral membrane proteins SIDT1 and SIDT2 are classified into a superfamily of putative metal-dependent hydrolases called CREST (alkaline ceramidase, PAQR receptor, Per1, SID-1, and TMEM8), in which alkaline ceramidase 3 and adiponectin receptors were reported to possess intrinsic ceramidase activity^3–5^. Moreover, SIDT1 and SIDT2 sharing sequence similarity with *Caenorhabditis elegans* cholesterol uptake protein 1 (CEL-CHUP-1) were revealed to participate in cellular cholesterol transport^6^. Notably, SIDT1 and SIDT2 also belong to the systemic RNA interference defective protein 1 (SID-1) family. Initially identified in *C. elegans* systemic RNAi^7^, SID-1 was proposed to be a dsRNA channel essential for importing silencing signals^8^. Further investigations on two human homologs hSIDT1 and hSIDT2 uncovered their critical roles in RNA binding, absorption, and transport. SIDT1 is responsible for dietary miRNA absorption from the stomach in a pH-dependent manner^9^. SIDT1 and SIDT2, located in the lysosomal or endolysosomal membrane, regulate innate immunity through RNA transport^10,11^. SIDT2 is also implicated in RNautophagy/DNautophagy^12^. These studies provided increasing evidence that SID-1 family proteins play critical roles in various biological processes and diseases. However, the underlying molecular mechanisms of RNA transport, cholesterol uptake, hydrolase activity, and their interconnections remain elusive.

Here, we determined the cryo-electron microscopy (cryo-EM) structures of hSIDT1 under pH 7.5 (hereafter hSIDT1^pH7.5^) and pH 5.5 (hereafter hSIDT1^pH5.5^) at 2.66 Å and 3.18 Å resolutions, respectively (Fig. 1a; Supplementary Figs. S1–S3 and Table S1), representing two distinct physiological states. Both hSIDT1^pH7.5^ and hSIDT1^pH5.5^ exhibit as homodimers, with each protomer having a large extracellular region and 11 transmembrane helices (TM1–11) (Fig. 1a, b). The extracellular region comprises two subdomains (ECD1, 2) with similar topology and contains 8 *N*-glycosylation sites, while the TMs are arranged systematically in a clockwise direction from the intracellular view, except for TM2 (Fig. 1b). Putative lipid densities surrounded by the transmembrane domain (TMD) were observed (Fig. 1a). hSIDT1^pH5.5^ has a slightly larger opening on the cytoplasmic side (Fig. 1a). The dimer interface is formed by both ECD and TMD, mainly through hydrogen bonds and hydrophobic interactions (Supplementary Fig. S4). In addition, 4 pairs of disulfide bonds were found in each protomer, enhancing intramolecular stability (Supplementary Figs. S4, S5).

**Fig. 1:**
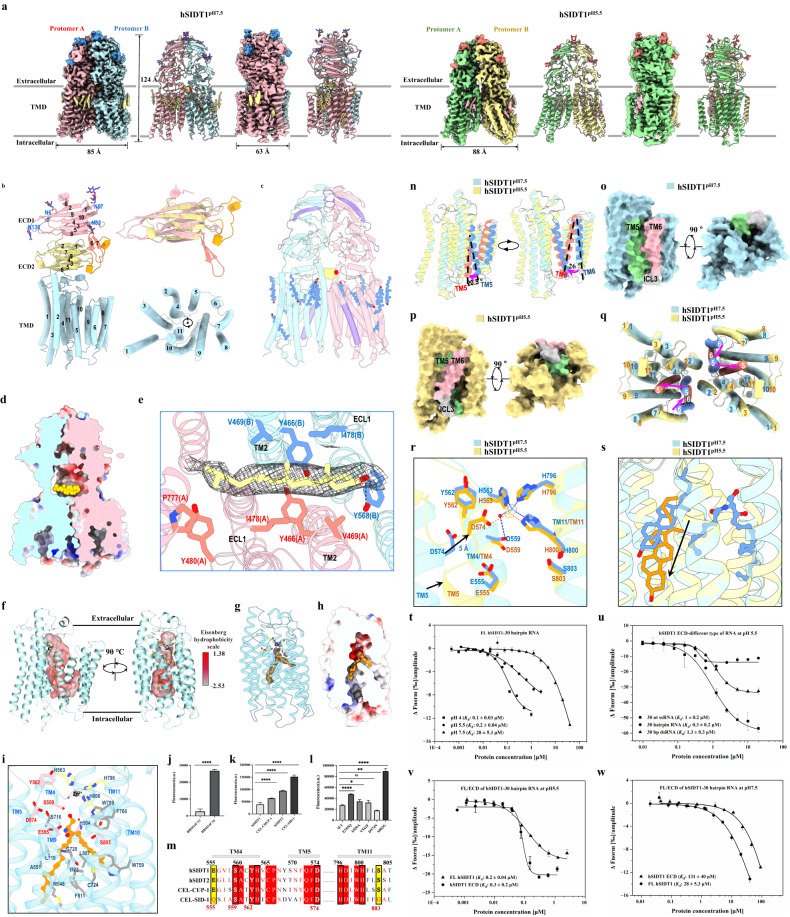
Structural and functional characterization of hSIDT1 under two distinct states. **a** The density maps and structural models of hSIDT1^pH7.5^ and hSIDT1^pH5.5^. In hSIDT1^pH7.5^, the lipids and glycosylations were colored yellow and royal blue, respectively. In hSIDT1^pH5.5^, they were in light pink and salmon, respectively. **b** Domain organization of hSIDT1 protomer. ECD1, ECD2, and TMD were colored light pink, yellow, and powder blue, respectively. **c** The cholesterol molecules at the dimer interface were shown as spheres and colored gold, while the cholesterols bound to the TMD surface were depicted as ball-and-stick in cornflower blue. The CRAC motifs were in medium purple. **d** The cholesterol molecule specifically bound to the extracellular juxta-membrane at the dimer interface. **e** The interaction between SIDT1 and cholesterol molecule. **f** A continuous cavity in the TMD, which was shown as surface and colored according to the Eisenberg hydrophobicity. **g** The putative Zn^2+^-binding site and the lipid density. **h** The lipid with two tails bound to the hydrophobic surface and polar head inserted into a negatively charged pocket. **i** Three histidine residues coordinating the putative Zn^2+^ were colored yellow, the critical polar residues were in light pink, and other involved residues were in gray. **j** Ceramidase activity of hSIDT1 with RBM14C12 and RBM14C16. **k** Ceramidase activity of hSIDT1 and its homologs. **l** Ceramidase activity of hSIDT1 mutants. **m** Structure-based sequence alignment of the residues around the putative Zn^2+^-binding site. **n** The TMDs of hSIDT1^pH7.5^ and hSIDT1^pH5.5^ protomer were superimposed, indicating shifts of TM5 and TM6. **o**, **p** The hSIDT1^pH7.5^ and hSIDT1^pH5.5^ protomers in open and closed forms, respectively. **q** The conformational change in TMD leads to the rearrangement of hSIDT1^pH5.5^ dimer interface. **r** The rearrangement of the putative Zn^2+^-binding site. Asp574 moves ~5 Å towards the putative Zn^2+^-binding site. **s** The shift of a cholesterol molecule, which was colored cornflower blue in hSIDT1^pH7.5^ and orange in hSIDT1^pH5.5^, as indicated by the black arrow. **t** Binding affinity of hSIDT1 with RNA under different pH values. **u** Binding affinity of hSIDT1-ECD with different types of RNA under pH 5.5. **v**, **w** Binding affinity of FL hSIDT1 and hSIDT1-ECD with 30 mer hairpin RNA under pH 5.5 (**v**) and pH 7.5 (**w**), respectively.

Previous studies suggested that SIDT1 is involved in cholesterol transport through two cholesterol recognition/interaction amino acid consensus sequence (CRAC) motifs^6^, located in ECD1 and TM7 in our structure (Fig. 1c). However, cholesterol-like densities were not identified near these motifs but rather observed in other distinct regions (Fig. 1c). A striking cholesterol-like density was observed in the extracellular juxta-membrane region at the dimer interface, which can be perfectly fitted by a cholesterol molecule (Fig. 1d, e). The binding of this cholesterol molecule is asymmetric, with the head hydroxyl group forming a hydrogen bond with Tyr568 from protomer B, the ring structure making contacts with Tyr466, Val469, and Ile478 from both protomers, and the hydrocarbon tail stabilized by Tyr480 and Pro777 from protomer A (Fig. 1e). Given the presence of cholesteryl hemisuccinate (CHS) in the buffer during membrane solubilization and the analogous core structure shared by CHS and cholesterol, this density can also be fitted by CHS (Supplementary Fig. S6a). Notably, other cholesterol-like densities are enriched at the hydrophobic surface of TMDs, forming extensive interactions with neighboring TMs and cholesterol molecules (Supplementary Fig. S6b, c and Discussion S1).

The hSIDT1 protomer harbors a putative Zn^2+^-binding site within the TMD close to the extracellular surface and a large internal cavity (Fig. 1f). The Zn^2+^ is coordinated by residues His563^TM4^, His798^TM11^, His800^TM11^ and a water molecule stabilized by residue Ser559^TM4^ (Fig. 1g, i). These residues are absolutely conserved throughout the CREST superfamily, suggesting that hSIDT1 should possess intrinsic ceramidase activity (Supplementary Fig. S7). Furthermore, a lipid-like density was observed in the internal cavity, which could accommodate an 8-carbon ceramide molecule (SPL; octanoic acid (2-hydroxy-1-hydroxymethyl-heptadec-3-enyl)-amide), forming extensive hydrophobic interactions with residues from TM3–5 and TM9–11 (Fig. 1f–i). We verified the ceramidase activity of hSIDT1 and its homologs using RBM14 fluorogenic ceramide analogs and found that hSIDT1 prefers long-chain acyl ceramide under the same experimental condition (Fig. 1j). CEL-SID-1 displayed the highest ceramidase activity, while there was no remarkable difference between hSIDT1 and CEL-CHUP-1, and the activity of hSIDT2 fell in between (Fig. 1k). Sequence alignment near the Zn^2+^-binding site suggested that the critical residues Ser559^TM4^, Tyr562^TM4^ and Asp574^TM5^ are strictly conserved, however, Glu555^TM4^ and Ser803^TM11^ vary to Gln and Gly in CEL-SID-1, respectively (Fig. 1m). Mutants S559A, E555Q, Y562F, D574N and S803G were generated for activity assay. As anticipated, the activity of E555Q and S803G significantly increases. Whereas S559A or Y562F does not display any noticeable difference compared to the wide-type (WT), and the activity of D574N slightly decreases (Fig. 1l; Supplementary Discussion S2).

Despite of similar domain organization, the structures of hSIDT1^pH7.5^ and hSIDT1^pH5.5^ assume distinct conformations. Significant changes occur in the TMD of each protomer, particularly in TM5 and TM6, which lead to an open form for hSIDT1^pH7.5^ and a closed form for hSIDT1^pH5.5^, triggering rearrangement at the dimer interface (Fig. 1n–q; Supplementary Discussion S3). Structural comparisons of hSIDT1 with its homologs revealed that the TMDs of these proteins adopt similar conformations to those of hSIDT1^pH5.5^ (Supplementary Fig. S8). The ECD of hSIDT1^pH5.5^ exhibits a nearly identical conformation to that observed in hSIDT1^pH7.5^ (Supplementary Fig. S9a).

The conformational changes in TMD have profound impacts on the arrangement of the catalytic center and binding state of cholesterols. In the hSIDT1^pH5.5^ protomer, the inward movement of TM5 results in the closure of the internal cavity, with no lipid density observed inside (Supplementary Fig. S9b). Notably, the Zn^2+^-binding site also undergoes rearrangement, wherein Asp574^TM5^ moves towards the Zn^2+^ ion, enabling direct coordination with it (Fig. 1r). The opening and closure of the internal cavity likely regulate substrate binding and product release (Supplementary Fig. S10). Additionally, the cholesterol density near the extracellular juxta-membrane in hSIDT1^pH7.5^ is absent in hSIDT1^pH5.5^, suggesting that cholesterol has already departed from this site at low pH. Interestingly, another cholesterol molecule, which binds close to the central cavity and interacts with SPL in hSIDT1^pH7.5^, undergoes a noticeable outward and downward movement (Fig. 1s; Supplementary Fig. S9c). The movement of this cholesterol molecule weakens its interaction with hSIDT1 (Supplementary Fig. S9d, e), indicating a state where the cholesterol molecule is poised to dissociate and potentially be released into the membrane.

The binding of hSIDT1 to RNA was suggested to be a prerequisite for RNA transport; we therefore conducted microscale thermophoresis (MST) assays to investigate the binding characteristics of hSIDT1 with small RNAs further. It was discovered that the binding of hSIDT1 with RNA is pH-dependent, exhibiting the highest affinity at pH 4 and gradually decreasing as the pH increases (Fig. 1t; Supplementary Fig. S11a, b). We found that the binding of hSIDT1 to RNA is also affected by the length (Supplementary Fig. S11d, e) and topological structures of RNA (Fig. 1u). Moreover, full-length hSIDT1 exhibits similar binding affinity as hSIDT1-ECD to hairpin RNA at low pH (Fig. 1v), but significantly higher affinity than hSIDT1-ECD at neutral pH (Fig. 1w; Supplementary Fig. S11c), indicating that hSIDT1 predominantly binds RNA through its ECD at pH 5.5. However, at neutral pH, other regions may also be involved in RNA binding. The surface charge distribution of hSIDT1 revealed two potential RNA-binding sites in both extracellular and intracellular regions (Supplementary Fig. S12a, b), reminiscent of RNA uptake into the lysosome by hSIDT2 through its cytosolic domain^12^. Remarkably, among the tested SIDT1 homologs, CEL-SID-1 displayed the highest binding affinity even at neutral pH, which is likely attributed to the increased charge density and expanded distribution in the positively charged regions of ECD (Supplementary Figs. S11f–h, S12c–f). The binding of hSIDT1 with dsRNA was further confirmed by small-angle X-ray scattering (SAXS) (Supplementary Fig. S13).

In this study, we determined the structures of hSIDT1 under two physiological pH conditions, revealing significant pH-dependent conformational changes that impact the dimeric interface, cholesterol-binding, catalytic centers, and RNA-binding properties of hSIDT1, implying interconnections among diverse functions (Supplementary Discussion S4). Given the absence of conventional channel features in the structures of hSIDT1^13,14^ and hSIDT2^15^, it is highly possible that these proteins employ a non-classical mechanism for translocating RNAs (Supplementary Fig. S14).

### Supplementary information

Supplementary information

## Data Availability

The atomic coordinates of non- and C2-symmetrized hSIDT1^pH7.5^ and hSIDT1^pH5.5^ have been deposited in the PDB database under the accession codes 8WOQ, 8WOR, 8WOS, and 8WOT, respectively. The corresponding EM density maps have been deposited in the EMDB database under the accession codes EMD-37695, EMD-37696, EMD-37697, and EMD-37698, respectively.
