# Supplementary material for: Structural insights into cholesterol transport and hydrolase activity of a putative human RNA transport protein SIDT1
Source: Cell Discov. 2024 Feb 20;10:21. doi: 10.1038/s41421-024-00647-2 (PMC10879482; doi:10.1038/s41421-024-00647-2)
Supplement: Supplementary file 1 — Supplementary information [file 41421_2024_647_MOESM1_ESM.pdf]

Supplementary information for

Structural insights into cholesterol transport and hydrolase activity of a putative human RNA transport protein SIDT1

Wenxia Liu[1, *], Mengyuan Tang[1, *], Jiening Wang[2, *], Fangfang Wang[3], Gaojie Song[3], Xiaokang Zhang[4, #], Shan Wu[2, #], Heng Ru[1, #]

[1]Zhejiang Provincial Key Laboratory for Cancer Molecular Cell Biology, Life Sciences Institute, Zhejiang University, Hangzhou, Zhejiang 310058, China

[2]State Key Laboratory of Biocatalysis and Enzyme Engineering, Hubei Collaborative Innovation Center for Green Transformation of Bio-Resources, Hubei Key Laboratory of Industrial Biotechnology, School of Life Sciences, Hubei University, 430062 Wuhan, China.

[3]Shanghai Key Laboratory of Regulatory Biology, Institute of Biomedical Sciences and School of Life Sciences, East China Normal University, Shanghai 200241, China.

[4]Interdisciplinary Center for Brain Information, The Brain Cognition and Brain Disease Institute，Shenzhen Institute of Advanced Technology, Chinese Academy of Sciences, Shenzhen, Guangdong 518055, China.

* These authors contribute equally to this work.

Co-correspondence to hengru@zju.edu.cn, wushan91@hubu.edu.cn, xk.zhang@siat.ac.cn

This PDF file includes:

Methods

Supplementary Discussions

14 Supplementary Figures

1 Supplementary Table

Methods

Protein production and purification

The coding sequence of full-length (FL) human SIDT1 (NP_001295279. 1,832 aa) was codon-optimized and subcloned into a modified BacMam vector with a C-terminal Strep and Flag tag. Constructs of hSIDT1 mutants including E555Q, S559A, Y562F, D574N, S803A were generated by transfer PCR. Besides, the coding sequence of hSIDT1 extracellular domain (ECD) was inserted into the vector pFastBac1 with a strep and flag tag at C-terminus. All constructs were verified by DNA sequencing. The resulting constructs were used to generate baculoviruses in *Spodoptera frugiperda* 9 (Sf9) insect cells according to the Bac-to-Bac instructions (Invitrogen). The HEK293F cells were infected with baculoviruses to produce FL hSIDT1 protein at a density of 3×10^6 cell/ml. After incubation at 37 °C for 12 h, the culture was supplemented by 10 mM sodium butyrate and transferred to 30 °C for another 36 h before harvest. Sf9 was infected by baculoviruses for secretory expression of ECD. The hSIDT1 mutants, the FL hSIDT1 homologues (FL hSIDT2 and CEL-SID-1 and CEL-CHUP-1) and their ECDs were produced by similar procedures.

For purification of FL proteins, cells were resuspended in buffer L (25 mM HEPES, 250 mM NaCl, 5% glycerol, pH 7.5) and disrupted by sonication (40% power, on 2 s, off 8 s, total 10 mins). After centrifugation at 2,000 g for 10 mins, the generated supernatant was further ultracentrifugated at 44,500 rpm (45 Ti rotor) for 1-2 h to prepare the membrane. The crude membrane was homogenized in buffer S (25 mM HEPES 500 mM NaCl, 10% Glycerol, pH 7.5 and protease inhibitors) by Dounce and solubilized using 1% (w/v) n-Dodecyl-β-D-Maltopyranoside (DDM, Anatrace) and 0.2% cholesteryl hemisuccinate (CHS; Anatrace) at 4 °C for 2 h. After centrifugation (35,000 rpm for 20 min), the supernatant was collected and incubated with preequilibrated Strep-Tactin Sepharose (smart-lifesciences) at 4 °C for 1 h. The incubated mixture was loaded into an empty gravity column, washed by buffer S and buffer L containing 0.01% glyco-diosgenin (GDN; Anatrace) sequentially. To elute the protein, 2.5 mM desthiobiotin (Sigma) was included in buffer L. Further protein purification was performed through size-exclusion chromatography (SEC) using a Superose 6 increase column (Cytiva). SEC buffer at pH 7.5

(25 mM HEPES, 100 mM NaCl, and 0.005% GDN), and pH 5.5 (25 mM MES, 100 mM NaCl, 0.005% GDN) were used to obtain hSIDT1 under different pH conditions. The purified hSIDT1 protein in the peak fractions was concentrated to approximately 10 mg/ml for cryo-EM studies.

To purify the ECD proteins, the cell culture medium containing each ECD was first clarified by centrifugation and then filtered through a 0.45 μM nitrocellulose membrane. The resulting supernatant was then incubated with strep beads at 4 °C for 1 hour. The resin was loaded into an empty gravity column and the contaminant protein was removed by buffer W1 (25 mM HEPES, 500 mM NaCl, pH7.5), followed by buffer W2 (25 mM HEPES, 100 mM NaCl, pH7.5). ECD was eluted from the column in buffer W2 containing 2.5 mM desthiobiotin. The eluted protein was further purified and homogenized by SEC using a Superose 6 increase column (Cytiva) in buffer containing either 25 mM HEPES, 100 mM NaCl at pH 7.5 or 25 mM MES, 100 mM NaCl at pH 5.5.

Cryo-EM sample preparation and data acquisition

Prior to cryo-EM sample preparation, an excess amount of 30 mer hairpin RNA and 30 bp dsRNA were incubated with hSIDT1 (in buffer at pH 5.5) at room temperature for 30 mins to generate hSIDT1-RNA complexes. For sample preparation, 3 μl hSIDT1 (purified under pH 7.5 and 5.5) and hSIDT1-RNA complex (pH 5.5) were applied onto glow discharged holey grids (Ni-Ti Au 300 mesh, NANODIM TECH), respectively. The grids blotted for 3 s under 100% humidity at 4 °C were rapidly plunged into liquid ethane (Vitrobot Mark IV (Thermo Fisher Scientific)). The cryo-EM data were collected at the Cryo-Electron Microscopy Facility of Hubei University. Micrographs were acquired on a 300 kV Titan Krios microscope with a BioQuantum energy filter. The movie stacks were collected on a K3 Summit detector (Gatan) in super-resolution counting mode (magnification of ×105,000, physical pixel size of 0.851 Å and dose rate of 15.156 e per pixel per second). The defocus range was set from -1.0 to -1.5 μm. The statistics of cryo-EM data collection are summarized in Supplementary Table S1.

Cryo-EM image processing

The original image stacks were imported into cryoSPARC (v4.2.1) for image processing. Motion correction was performed using Patch motion correction, and contrast transfer function (CTF) estimation was done by patch CTF estimation. Micrographs with an estimated CTF resolution of lower than 4 Å were discarded during the manual curation. Initial particle picking was performed using the blob picker, and final picking was done by template picker with a template generated from the initial 3D volume. Particles were then extracted with a box size of 360 pixels and cropped into 180 pixels to accelerate early-step calculation. The resulting particles underwent two-dimensional (2D) classification to remove obvious junk particles. The remaining particles were subjected to *ab initio* reconstruction and 3D refinement.

For hSIDT1$^{pH7.5}$, a total of 12,083 micrographs were collected. After the Manually Curate Exposures step, 11,852 micrographs were selected for particle picking. The resulting 10,241,329 particles were further subjected to several rounds of 2D classification to remove trash particles. *Ab initio* reconstruction on 745,618 particles from selected 2D averages generated four classes. 627,880 good particles (84.2%) yield from three classes were subjected to heterologous refinement. Two good classes displaying obvious secondary structure features were selected for non-uniform refinement, resulting in resolution of 3.88 Å and 2.97 Å, respectively. The electron density maps from these two classes were identical, so the particles from each class were combined and re-extracted using a box size of 360 pixels, resulting in 503,286 particles. After global and local CTF refinement, the particles were subjected to non-uniform refinement, yielding a final resolution at 2.85 Å. The same set of particles was imposed with C2 symmetry, resulting in a symmetrized map at 2.66 Å resolution.

For hSIDT1$^{pH5.5}$ and hSIDT1-RNA complex, similar procedures were followed, with slight modifications. 13,483 micrographs were selected out of 13,862 collected movies for particle picking. The resulting 13,972,342 particles underwent several rounds 2D classification to discard the junk particles. 317,674 particles from selected 2D average were used for *ab initio* reconstitution. Heterologous refinement was carried out on all three classes generated from the previous step. The class with best resolution (46.5%, 5.89 Å)

was used for non-uniform refinement, resulting in a resolution at 3.45 Å. The particles from this class were re-extracted for non-uniform refinement with C2 symmetry, resulting in a resolution at 3.31 Å. The resulting particles were further subjected to non-uniform refinement after global and local CTF refinement. The final resolution was improved to 3.18 Å. Unexpectedly, no RNA densities were observed in either the 2D class averages or the final EM maps, we reasoned that the absent of RNA densities might due to frozen damage or dissociation of the complex at the air-liquid interface.

Atomic model building and refinement

The high-quality hSIDT1$^{pH7.5}$ map enabled automated building of an initial atomic model using Model Angelo [1]. For hSIDT1$^{pH5.5}$, the model was manually built in COOT [2], guided by the AlphaFold-predicted hSIDT1 structure docked into the EM density in Chimera [3]. Further manual adjustments were performed using COOT. The final models were refined in real space using PHENIX [4]. Local resolutions were estimated by cyroSPARC. All structural figures were generated using PyMOL [5], Chimera, and Chimera X [6]. The data validation and model refinement statistics are summarized in Supplementary Table S1.

Ceramidase activity assay

The ceramidase activity assay of wild type hSIDT1, hSIDT1 mutants (E555Q, S559A, Y562F, D574N, S803A) and its homologous proteins including hSIDT2, CEL-SID-1 and CEL-CHUP-1 were determined according to a previously reported protocol using fluorogenic ceramide analogs RBM14 compound as the substrate [7]. Briefly, the assays were carried out in 96-well plates with a final volume of 100 µL/well. Purified protein (0.5 µM) was incubated with RBM14C12 or RBM14C16 (40 µM) in a reaction buffer containing 25 mM HEPES, 150 mM NaCl, and 0.005% GDN, pH7.5 at 37°C for 3 hours without agitation. A reaction mixture without protein was used as a blank. The enzymatic reaction was stopped by adding 50 µl methanol and 100 µl freshly prepared 2.5 mg/ml $NaIO_4$ solution in 100 mM glycine/NaOH buffer (pH 10.6) to each well. The plate was protected from light for 1 hour and then the released fluorescence was measured using a microplate fluorescence reader (Ex 345 nm, Em 460 nm). The reaction buffers used for activity assay under different pH were as follows: 25 mM sodium acetate, 150 mM NaCl

at pH 4.0, 25 mM MES, 100 mM NaCl at pH 5.5, 25 mM HEPES, 150 mM NaCl at pH 7.5 and 25 mM CHES, 150 mM NaCl at pH 9.0, respectively. All reaction buffers contained 0.005% GDN. Statistical analyses were performed by GraphPad (https://www.graphpad.com/quickcalcs/ttest2/). Ordinary one-way ANOVA was used to determine statistical significance. ****$P < 0.0001$.

Microscale thermophoresis (MST) assay

MST experiments were performed using a Monolith NT.115 instrument (NanoTemper Technologies, Germany) at room temperature. Binding buffers under different pH values (25 mM HEPES, 100 mM NaCl, 0.005% GDN at pH 7.5, 25 mM MES, 100 mM NaCl, 0.005% GDN at pH 5.5 and 25mM sodium acetate acid, 100 mM NaCl, 0.005% GDN at pH 4.0) were used. The experiments were carried out at 25 °C. For the protein-RNA binding assay, RNA oligos labeled with 5'-Alexa 488 fluorophore were synthesized by Generay. The 5'-Alexa 488 labeled RNA was diluted to a concentration of 40 nM in binding buffer containing 0.01% Tween 20. It was then incubated with an equal volume of a serial dilutions of protein sample at room temperature for 30 mins prior to measurement. MST data was analyzed by NanoTemper Analysis 1.2.101 software and Origin Lab. The data were fitted with the K_d model to determine the dissociation constant. Each experiment was technically repeated at least three times (n = 3), and the K_d values were calculated with standard deviation and error bars represent the means ± SD.

Small-angle X-ray scattering (SAXS)

Small-angle X-ray scattering (SAXS) experiments were performed at beamline BL19U2 of National Facility for Protein Science Shanghai (NFPS) at Shanghai Synchrotron Radiation Facility (SSRF). The wavelength (λ) of X-ray radiation was set as 0.918 Å. Scattered X-ray intensities were collected using a Pilatus 1M detector (DECTRIS Ltd). The sample-to-detector distance was set such that the detecting range of momentum transfer [$q = 4\pi \sin\theta/\lambda$, where 2θ is the scattering angle] of SAXS experiments was 0.008-0.47 $Å^{-1}$. To reduce the radiation damage, a flow cell made of a cylindrical quartz capillary with a diameter of 1.5 mm and a wall of 10 μm was used. SAXS data were collected as 20 × 1 sec exposures and scattering profiles for the 20 passes were compared at 10 °C using 60 μL sample in buffer containing 25 mM MES, pH 5.5, 100 mM NaCl, and 0.005% GDN.

Measurements were carried out at two different concentrations in all cases using concentrations between 1.5 and 3 mg/ml. Background scattering was subtracted using PRIMUS in ATSAS software package [8]. Pair distance distribution functions of the particles *P(r)* and the maximum sizes D_{max} were computed using GNOM [6]. The *ab initio* structural models were reconstructed using DAMMIF [9]. The envelopes are aligned, clustered, and averaged using DAMAVER [10].

Supplementary Discussion

1. The diverse and intricate cholesterol binding patterns observed in hSIDT1 imply its potential roles in cholesterol transport and homeostasis.

2. The enhanced activity observed in the mutant E555Q may be attributed to alterations in substrate binding specificity or recognition. While the mutation of S803G likely increases the flexibility of TM11, where residues His798 and His800 are situated.

3. In hSIDT1$^{pH5.5}$, TM5 undergoes a remarkable inward displacement up to 20.5° and TM6 concomitantly tilts by approximately 26°, making the entire monomer more compact. These two helices are involved in the constitution of the central cavity of each protomer as well as the formation of dimer interface, hence establishing a dynamic correlation between the monomer and dimer conformational changes. Such elaborate design and arrangement provide structural foundations for SIDT1 to carry out multiple functions.

4. hSIDT1, hSIDT2, CEL-SID-1 and CEL-CHUP-1 proteins all belong to the SID-1 family, exhibiting RNA transport and ceramidase activity, but differing in enzymatic activity levels and RNA-binding capabilities. These proteins have diverged in cellular distribution and physiological functions during evolution. It was suggested by Christian Wolfrum *et al* that cellular uptake of cholesterol-conjugated siRNAs requires the mammalian homolog of the CEL-SID-1[11]. In addition, dsRNA transport of SIDT1 can be attained when mixed with cholesterol[12]. Strikingly, the import of dsRNA was impaired for mutants S536I, H740Y and G747R in CEL-SID-1[13]. While residues Ser536, His740 and Gly747 in CEL-SID-1 correspond to Ser559, His796 and Ser803 in hSIDT1, which are all located in the ceramidase catalytic center. Moreover, our biochemical assays indicated that CEL-SID-1, possessing highest ceramidase activity over its homologues, also exhibits the strongest binding affinity to RNA. These different lines of studies provide certain evidences for the important roles of cholesterol transport and ceramidase activity in RNA transport mediated by SID-1 family proteins.

Together, our comprehensive structural studies and functional assays expand our understanding about the multiple roles of the SID-1 family proteins and bridge the seemingly unrelated three functional aspects of hSIDT1. This convergence of knowledge

represents a pivotal step forward in unraveling the intricate mechanisms underlying the diverse functions of the SID-1 family proteins.

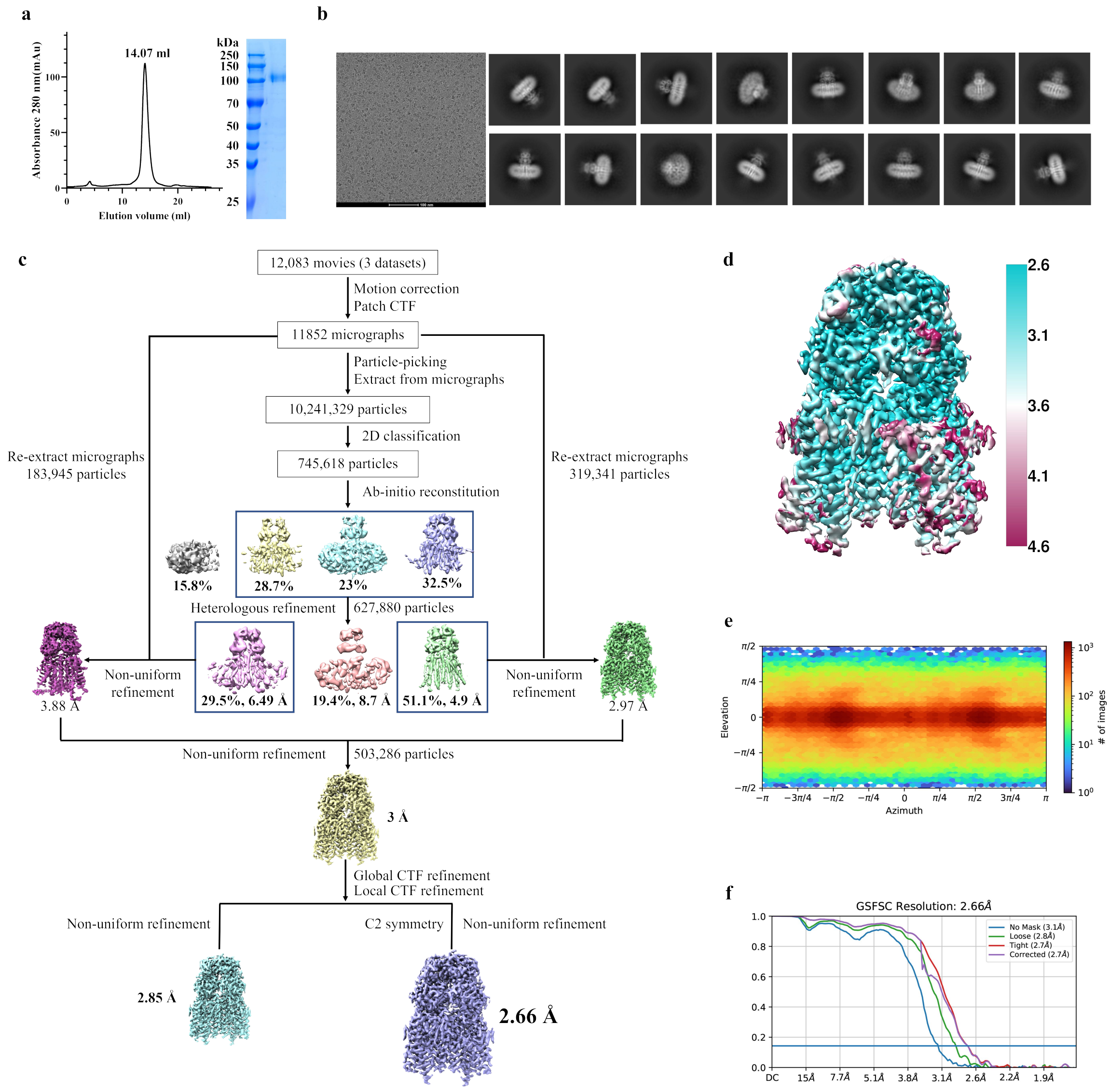

Supplementary Fig. S1 | Structure determination of hSIDT1$^{pH7.5}$. a, Size-exclusion chromatography of hSIDT1$^{pH7.5}$ on a Superose 6 increase column (Cytiva) and SDS-PAGE analysis of peak fraction. **b**, Representative cryo-EM micrograph and 2D class averages of hSIDT1$^{pH7.5}$. **c**, Cryo-EM data processing workflow for hSIDT1$^{pH7.5}$. **d**, The density map of hSIDT1$^{pH7.5}$ colored by local resolution (Å). **e**, Angular distribution plot of particles included in the final C2-symmetric 3D reconstruction of hSIDT1$^{pH7.5}$. **f,** Gold-standard Fourier shell correlation curves for electron microscopy map of hSIDT1$^{pH7.5}$.

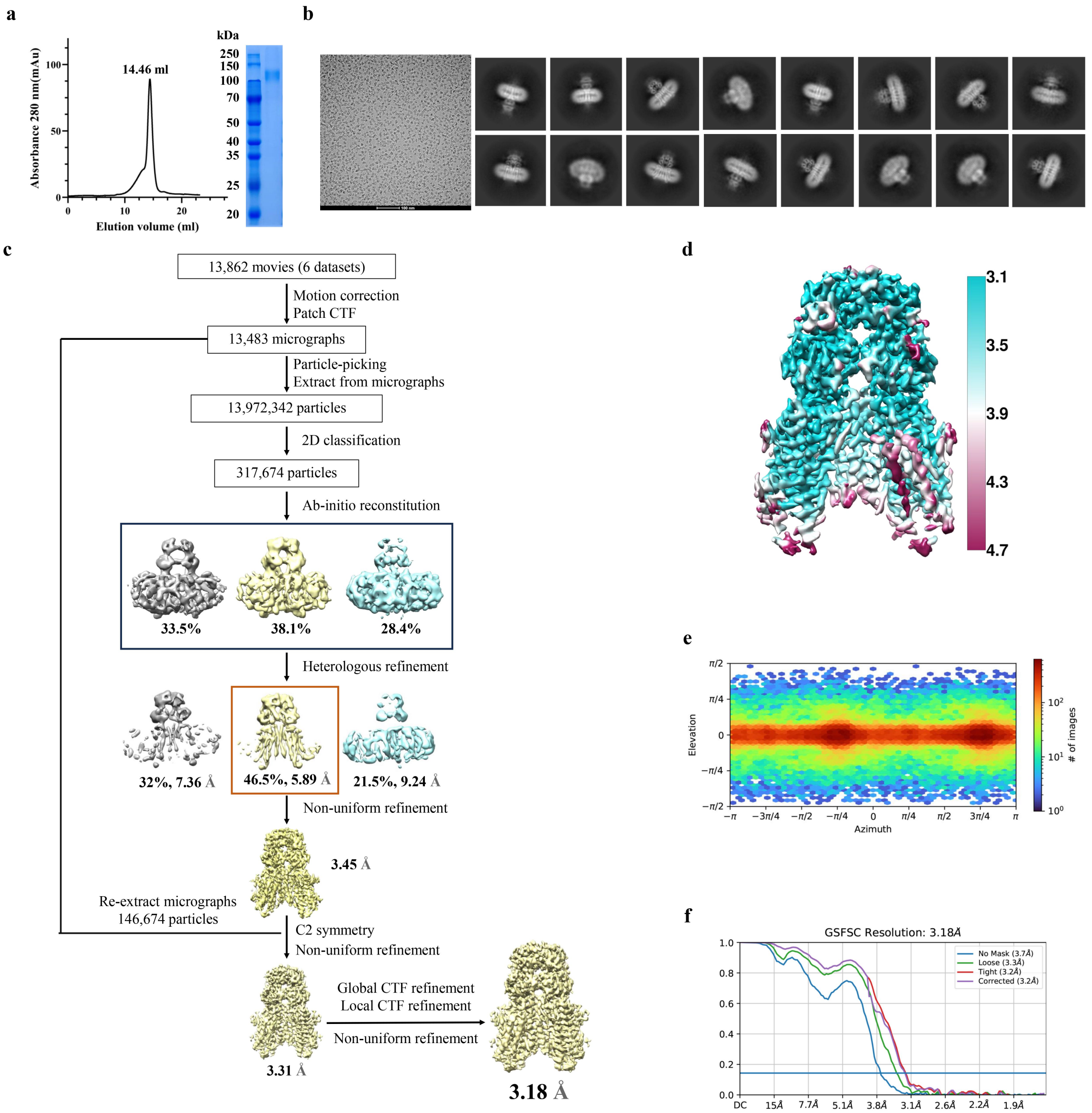

Supplementary Fig. S2 | Structure determination of hSIDT1$^{pH5.5}$. **a**, Size-exclusion chromatography of hSIDT1$^{pH5.5}$ on a Superose 6 increase column (Cytiva) and SDS-PAGE analysis of peak fraction. **b**, Representative cryo-EM micrograph and 2D class averages of hSIDT1$^{pH5.5}$. **c**, Cryo-EM data processing workflow for hSIDT1$^{pH5.5}$. **d**, The density map of hSIDT1$^{pH5.5}$ colored by local resolution (Å). **e,** Angular distribution plot of particles included in the final *C2*-symmetric 3D reconstruction of hSIDT1$^{pH5.5}$. **f,** Gold-standard Fourier shell correlation curves for electron microscopy map of hSIDT1$^{pH5.5}$.

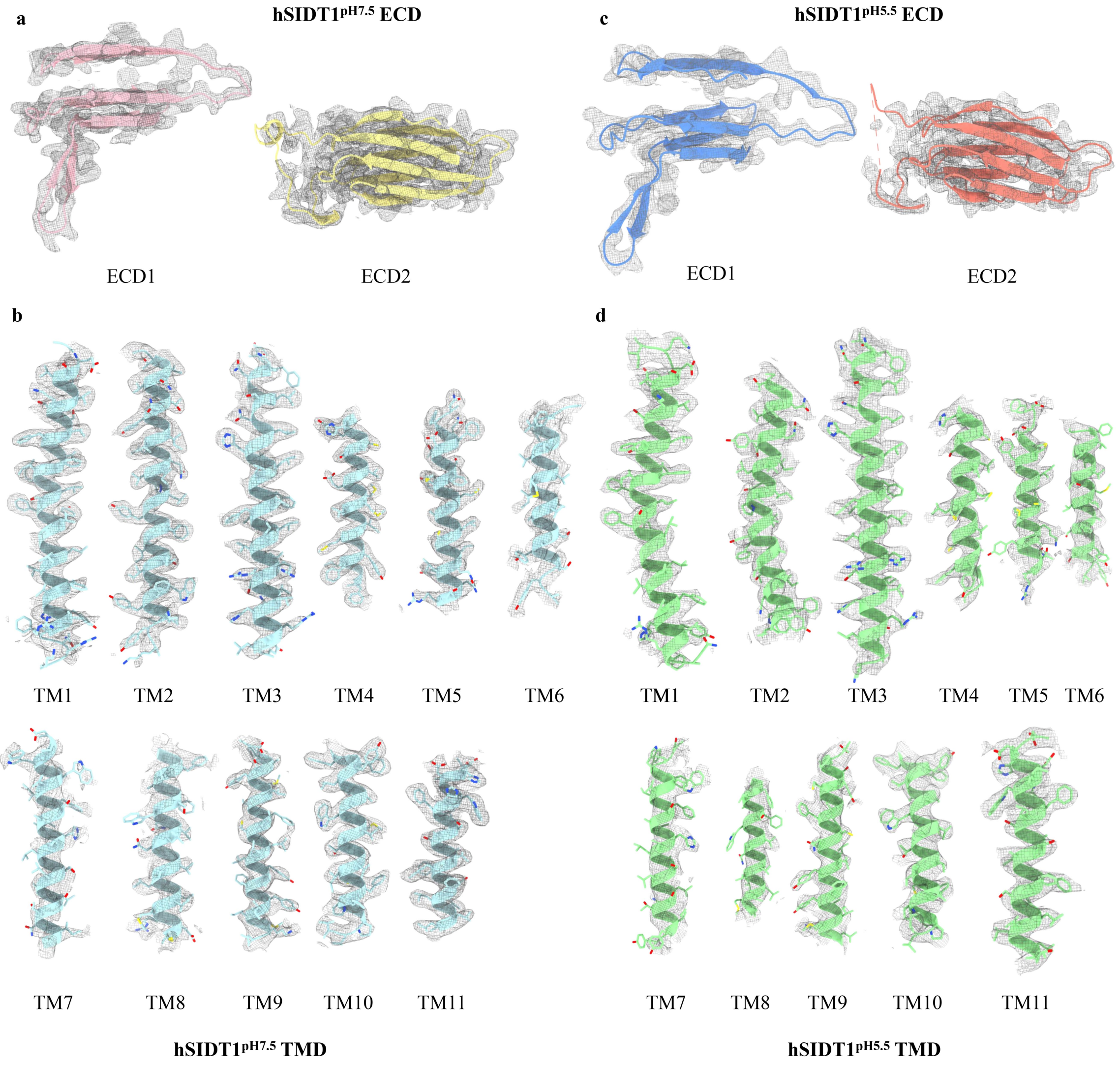

Supplementary Fig. S3 | Representative local cryo-EM maps of hSIDT1$^{pH7.5}$ and hSIDT1$^{pH5.5}$. a, The EM densities of hSIDT1$^{pH7.5}$ ECD. **b,** The EM densities of hSIDT1$^{pH7.5}$ TMD. **c,** The EM densities of hSIDT1$^{pH5.5}$ ECD. **d,** The EM densities of hSIDT1$^{pH5.5}$ TMD.

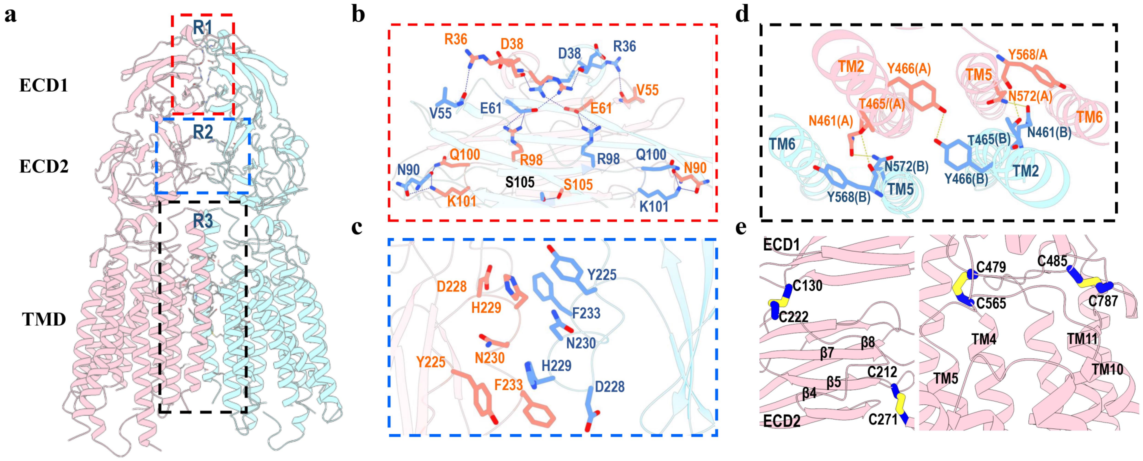

Supplementary Fig. S4 | Dimer interface. a, The dimer interface can be divided into three regions: R1, R2 and R3, which was boxed in red, blue and black dashes, respectively. **b,** R1 was mediated by hydrophobic interactions and hydrogen bonds. The hydrogen bonds were indicated as black dashed lines. **c,** R2 was formed by hydrophobic interactions. The residues mediating hydrophobic contacts were displayed. **d,** R3 was engaged by extensive hydrophobic interaction and several hydrogen bonds. The involved hydrogen bonds were displayed. **e,** Intramolecular disulfide bonds. There are four pairs of disulfide bonds colored in blue. The first pair was connected by Cys130 from ECD1 and Cys222 from ECD2. The second one was linked by Cys212 and Cys271 in ECD2. The third and fourth one was paired by Cys479 and Cys565, Cys485 and Cys787, which linked the loop between TM4 and TM5, and TM10 and TM11, respectively.

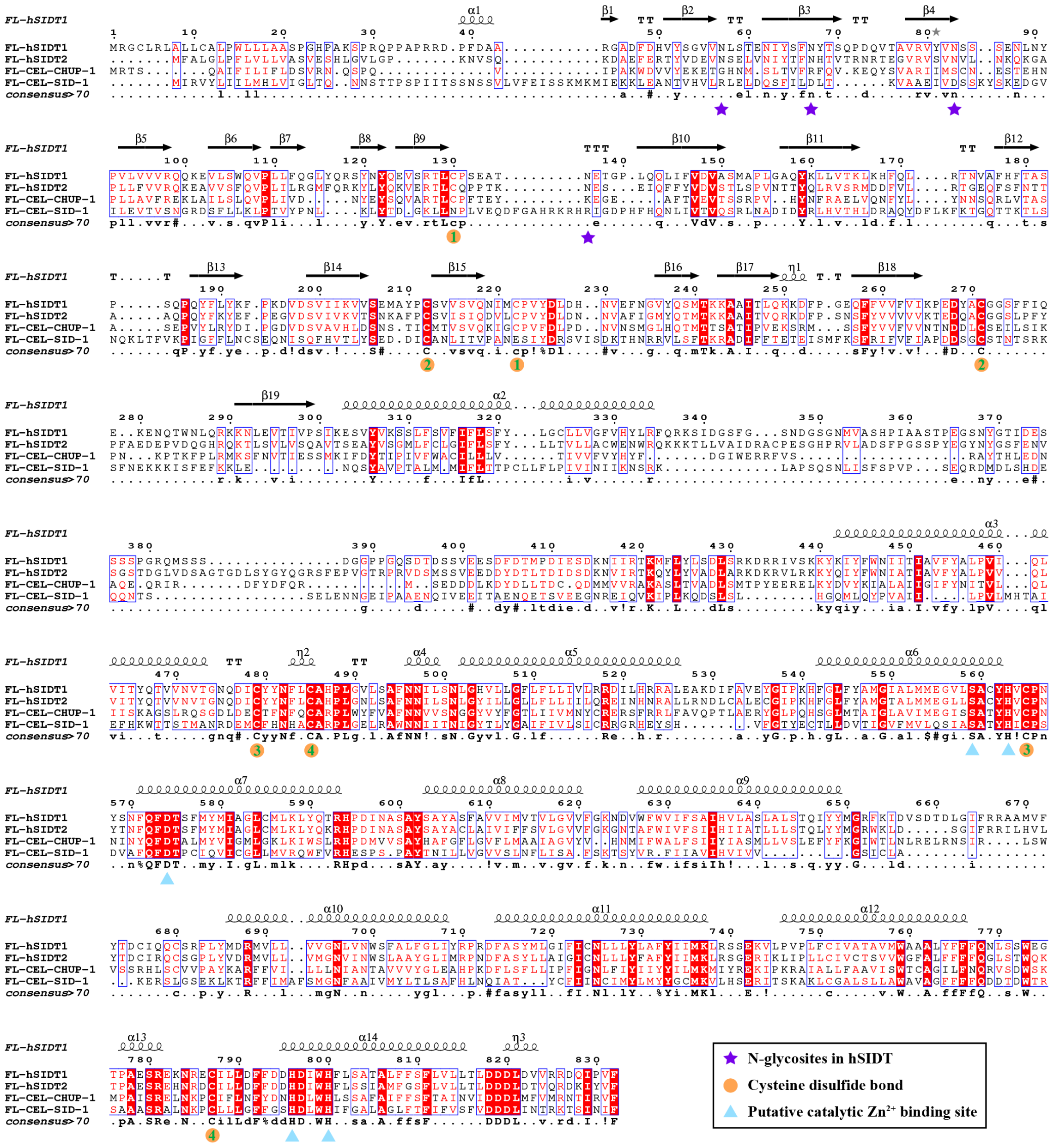

Supplementary Fig. S5 | Structure-based sequence alignment of hSIDT1 and its homologues. The sequence alignment was performed using Clustal Omega (https://www.ebi.ac.uk/Tools/msa/clustalo/) and visualized by ESPript 3.0 (https://espript.ibcp.fr/ESPript/ESPript/). The secondary structural elements of hSIDT1 were indicated above the sequence alignment. Red shading was used to highlight identically conserved residues. The four pairs of disulfide bonds were represented by orange circles, which are absolutely conserved across the SID-1 family with exception for the first pair of disulfide bond formed by Cys130 and Cys222. Additionally, the N-

glycosylation sites in hSIDT1 are indicated by violet pentacles. The residues coordinating the putative Zn^{2+} were represented by cyan triangles.

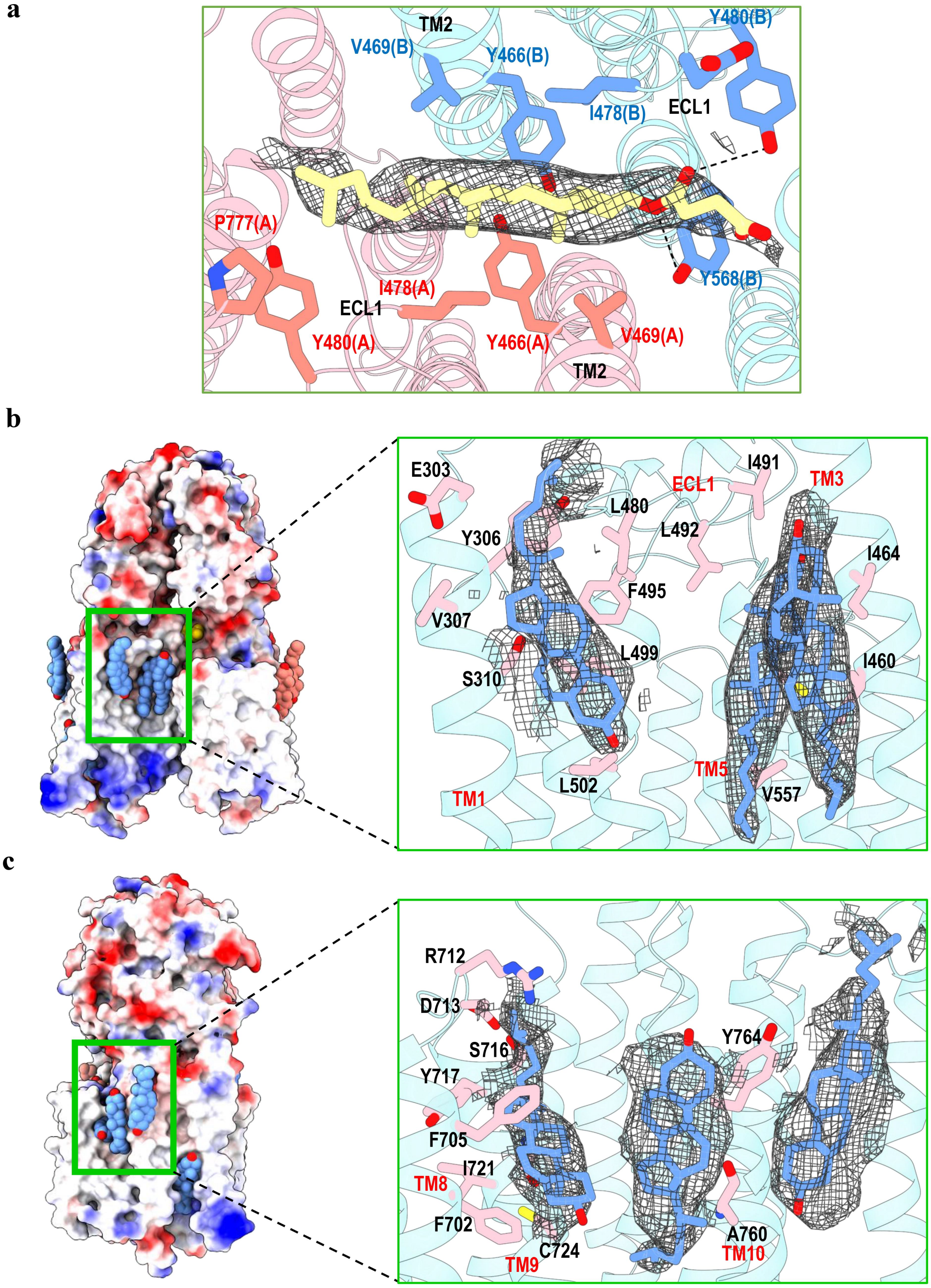

a
TM2
V469(B)
Y466(B)
Y480(B)
I478(B)
ECL1
P777(A)
I478(A)
ECL1
Y480(A)
Y466(A)
V469(A)
TM2
Y568(B)
b
E303
ECL1
I491
TM3
L480
Y306
L492
I464
F495
V307
L499
I460
S310
TM5
TM1
L502
V557
c
R712
D713
S716
Y764
Y717
F705
I721
TM8
F702
C724
TM9
A760
TM10

Supplementary Fig. S6 | Cholesterol binding modes. a, CHS was fitted in the cholesterol-like density at the dimer interface. **b and c,** A cluster of cholesterol molecules form extensive interactions with the residues of neighboring TMs and lipids, which are colored by cornflower blue. (b) Cholesterol molecules are bound to the hydrophobic pocket formed by TM1, 3, 5 and ECL1 connecting TM2 and TM3. (c) Cholesterol molecules are bound to the hydrophobic surface formed by TM8, 9 and 10. The involved transmembrane regions were labelled and the engaged residues are depicted as sticks and colored by light pink.

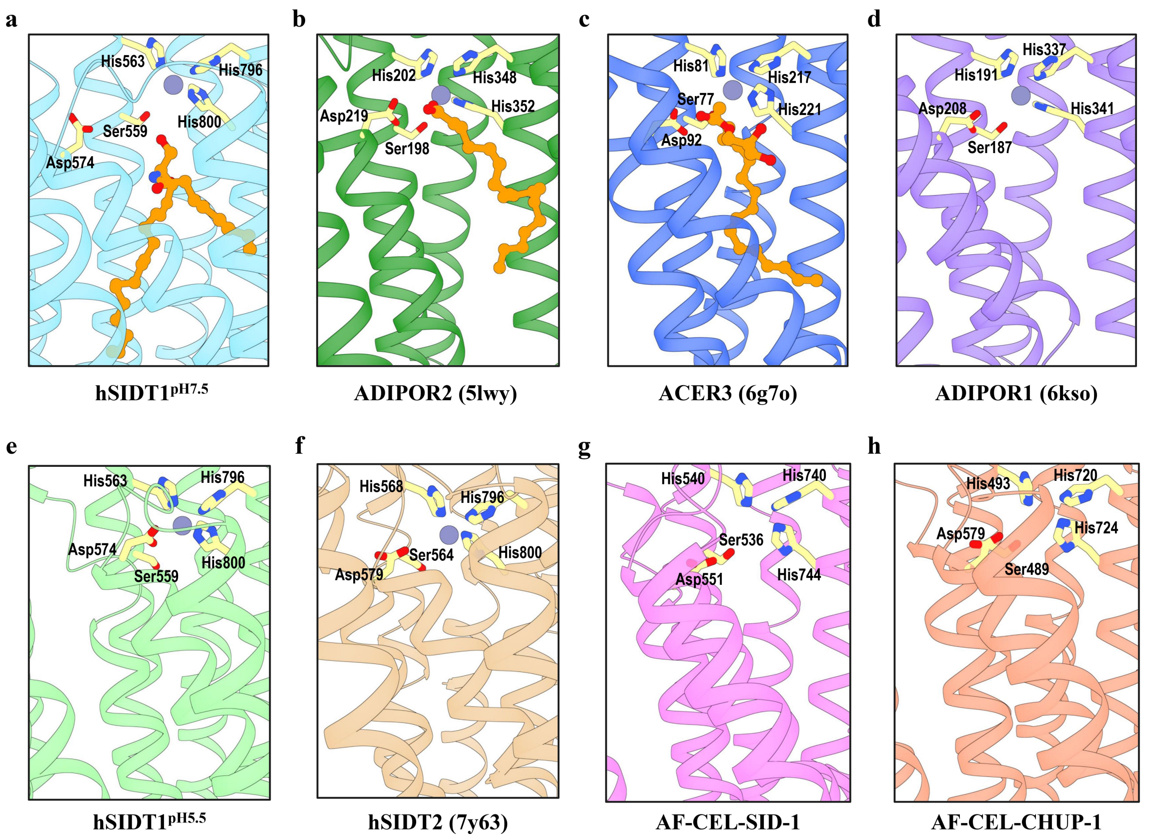

Supplementary Fig. S7 | Structural features of the Zn^{2+}-dependent active center of hydrolase shared by CREST superfamily protein. The putative Zn^{2+} is coordinated by three absolutely conserved histidine residues, which are shown as sticks and colored by yellow. The Zn^{2+} is depicted as a sphere and colored in grey. **a,** The putative Zn^{2+}-binding site in the structure of hSIDT1$^{pH7.5}$. There is a lipid bound to the central cavity, which may serve as the substrate or substrate analog. **b,** The putative Zn^{2+}-binding site in the structure of ADIPOR2. A fatty acid molecule is present in the internal cavity. **c,** The putative Zn^{2+}-binding site in the structure of ACER3. A monoolein molecule displays in the internal cavity. **d,** The putative Zn^{2+}-binding site in the structure of ADIPOR1. **e,** The putative Zn^{2+}-binding site in the structure of hSIDT1$^{pH5.5}$. There is no lipid binding in the cavity. **f,** The putative Zn^{2+}-binding site in the structure of hSIDT2 (7y63). No lipid was observed. **g,** The putative Zn^{2+}-binding site in the structure of CEL-SID-1 predicted by Alphafold2. **h,** The putative Zn^{2+}-binding site in the structure of CEL-CHUP-1 predicted by Alphafold2. The putative Zn^{2+} was not depicted in the predicted structure.

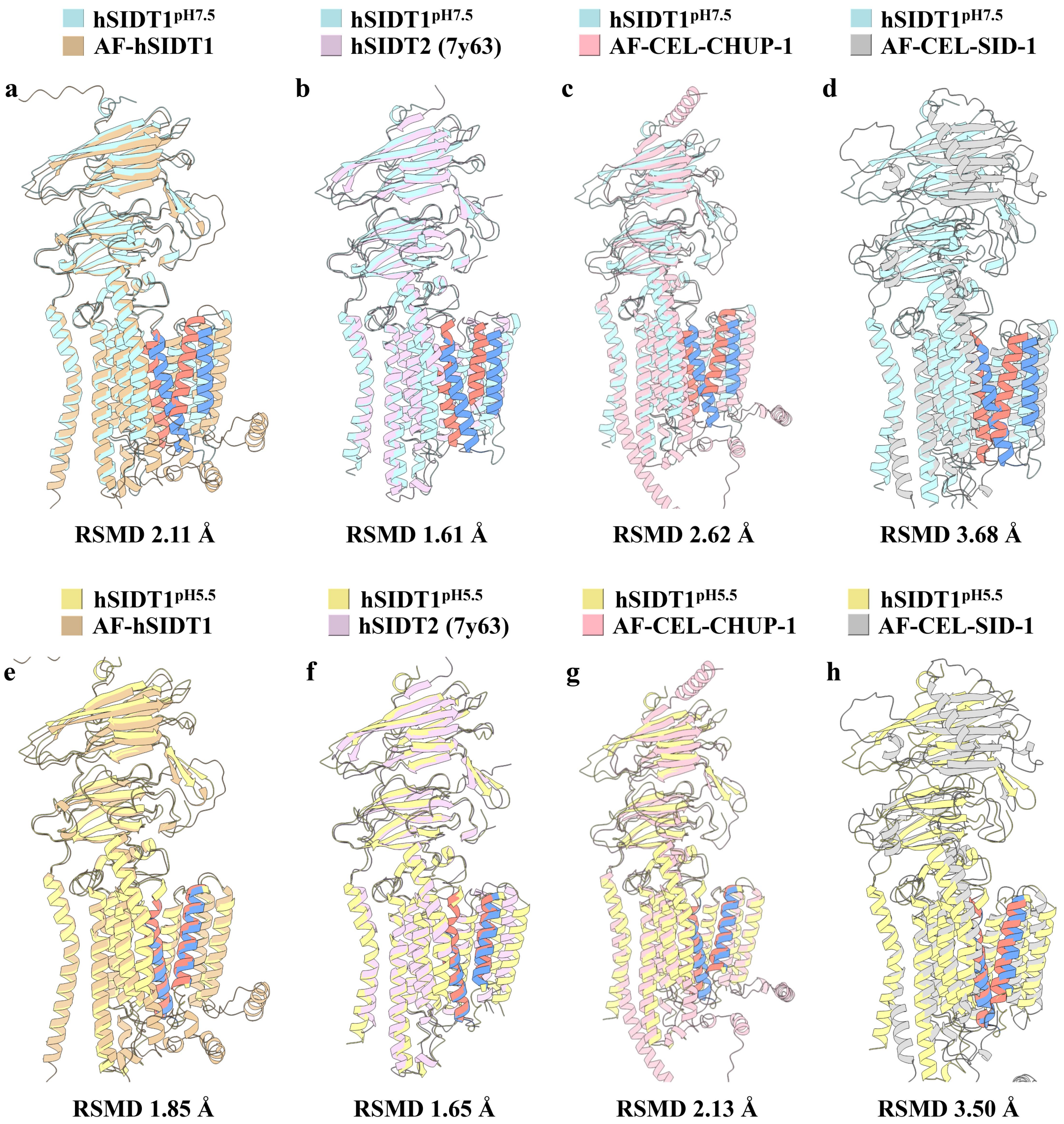

Supplementary Fig. S8 | Structural comparisons of hSIDT1 to AF-hSIDT1, hSIDT2, AF-CEL-CHUP-1, and AF-CEL-SID-1. hSIDT1$^{pH7.5}$ was colored by powder blue, while hSIDT1$^{pH5.5}$ was colored by yellow. AF-hSIDT1, hSIDT2 (7y63), AF-CEL-CHUP1 and AF-CEL-SID-1 were colored by tan, thistle, light pink, and silver, respectively. TM5 and TM6 were highlighted by cornflower blue in the structure of hSIDT1 and by salmon in its homologues. **a-d,** The structure of AF-hSIDT1, hSIDT2, AF-CEL-CHUP-1, and AF-CEL-SID-1 were superimposed to hSIDT1$^{pH7.5}$, respectively. **e-h,** The structure of AF-hSIDT1, hSIDT2, AF-CEL-CHUP-1, AF-CEL-SID-1 were superimposed to hSIDT1$^{pH5.5}$, respectively. The TMDs of AF-hSIDT1, hSIDT2, AF-CEL-CHUP-1, and AF-CEL-SID-1

TMD adopt similar conformation to hSIDT1$^{pH5.5}$, but distinct from hSIDT1$^{pH7.5}$, particularly in TM5 and TM6.

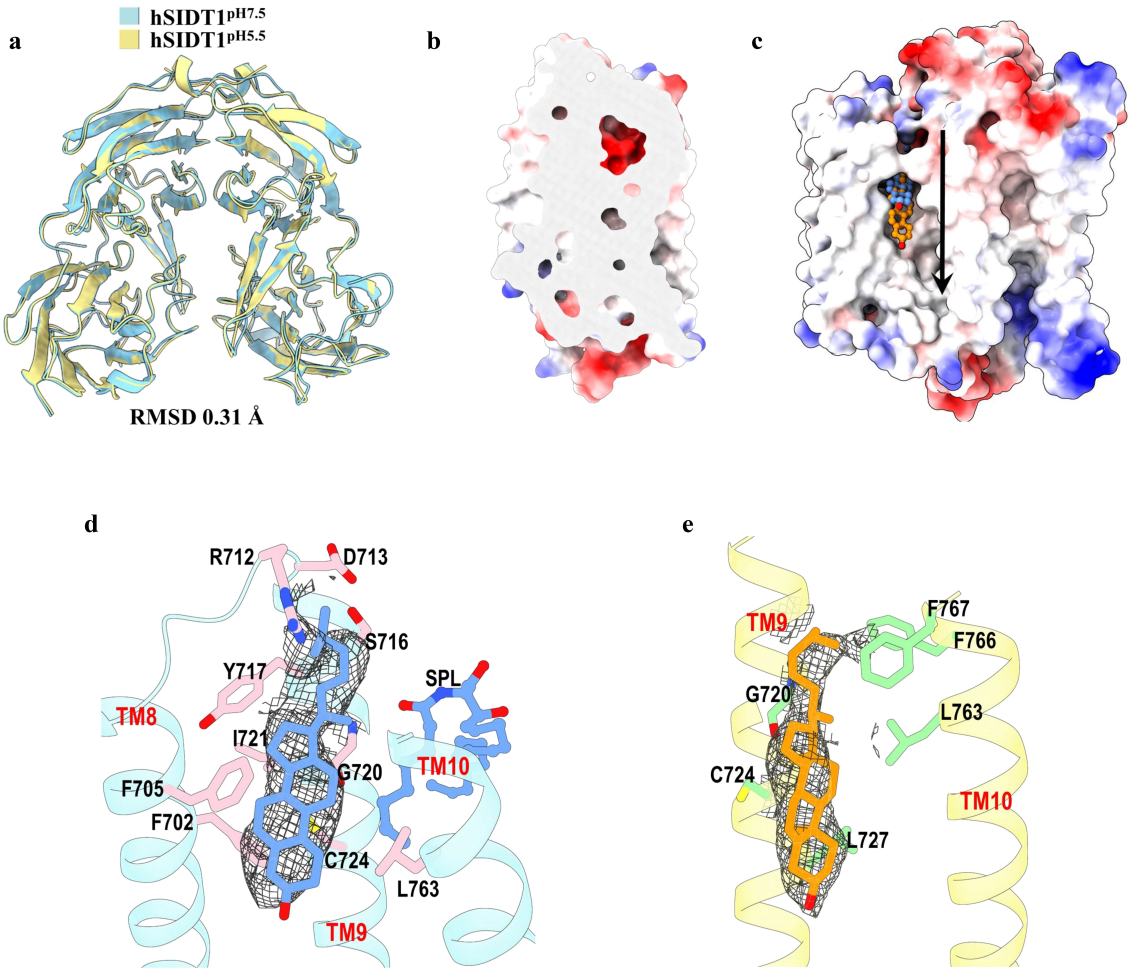

Supplementary Fig. S9 | Structural comparison of hSIDT1$^{pH7.5}$ and hSIDT1$^{pH5.5}$ ECD and the pH-dependent variations in cholesterol binding state and the catalytic center. **a,** The ECD of hSIDT1$^{pH7.5}$ and hSIDT1$^{pH5.5}$ exhibited nearly identical conformations, with an RMSD of 0.31 Å. **b,** In hSIDT1$^{pH5.5}$ TMD, the central cavity was closed, with no lipid observed inside. **c-e,** The shift of a cholesterol molecule observed in the structure hSIDT1$^{pH5.5}$. (c) The TMD of hSIDT1$^{pH7.5}$ and hSIDT1$^{pH5.5}$ were superimposed and the displacement of this cholesterol from the hydrophobic binding pocket was indicted by black arrow. (d) This cholesterol molecule binds near the central cavity in the structure of hSIDT1$^{pH7.5}$, forming extensive interactions with residues from TM8, 9, 10 and the SPL

molecule. (e) The interaction between this cholesterol and the nearby TMs was weakened in the structure of $hSIDT1^{pH5.5}$.

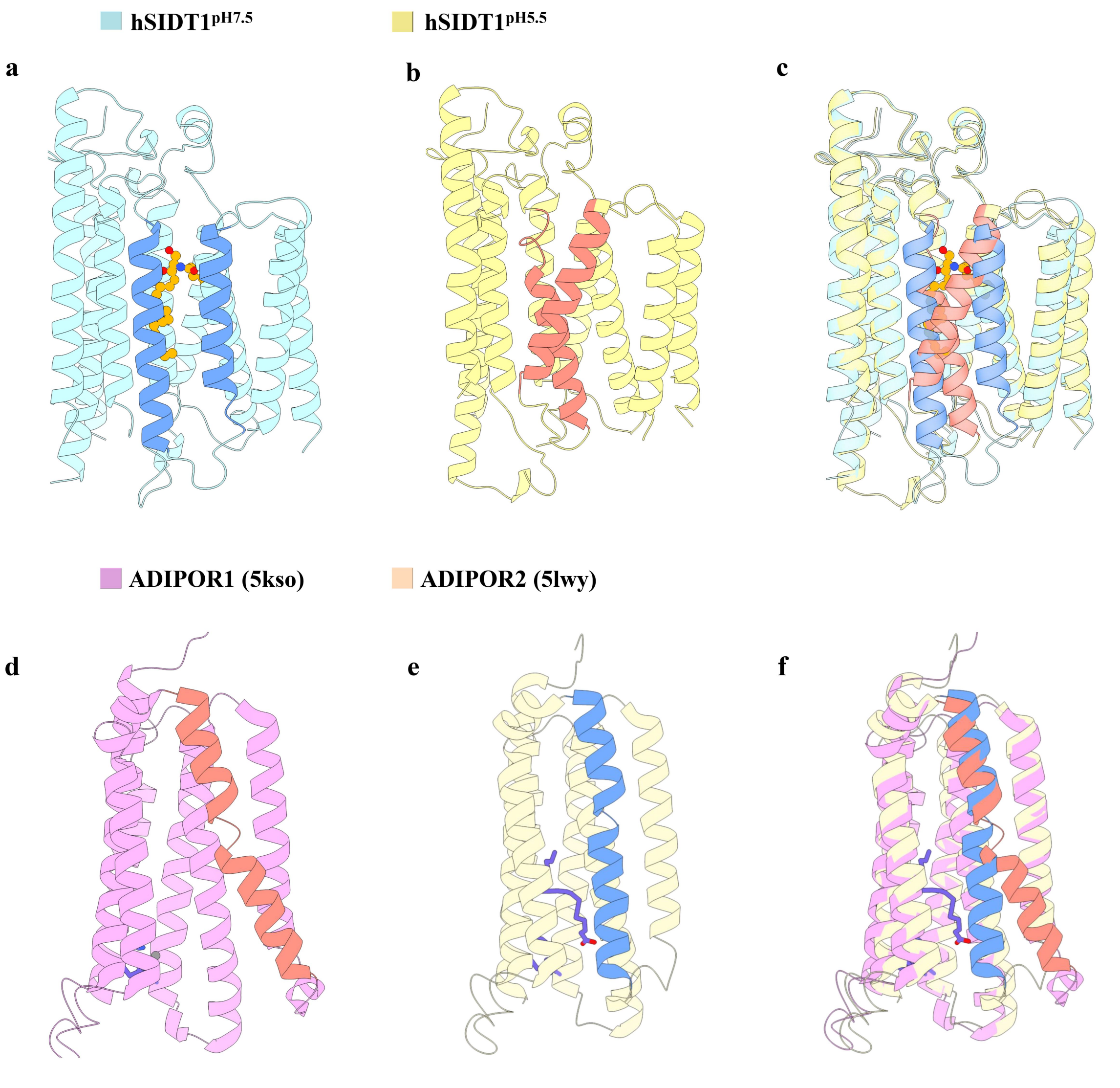

Supplementary Fig. S10 | Comparison of the open and closed form of hSIDT1 and ADIPORs. a, The open form of hSIDT1 under pH 7.5, with a ceramide (SPL) bound to the central cavity. **b,** The closed form of hSIDT1 under pH 5.5, without any lipid bound near the Zn^{2+}-binding site. **c,** When the TMDs of $hSIDT1^{pH7.5}$ and $hSIDT1^{pH5.5}$ are superimposed, two critical transmembrane helices TM5 and TM6 undergo dramatic conformational change, modulating the opening and closure of the internal cavity of hSIDT1. The two helices were colored by cornflower blue in $hSIDT1^{pH7.5}$ and by salmon

in hSIDT1$^{pH5.5}$. **d,** The open form of ADIPOR1, without lipid in the central cavity. **e,** The closed form of ADIPOR2, with an oleic acid molecule bound near the Zn^{2+}-binding site. **f,** Superimposing the TMD of ADIPOR1 with ADIPOR2 revealed the tilt of TM5. The transition between the open and closed conformation of hSIDT1 protomer resembles that of ADIPOR1 and ADIPOR2, but it appears that the open and closed forms of hSIDT1 and ADIPOR represent distinct catalytic steps.

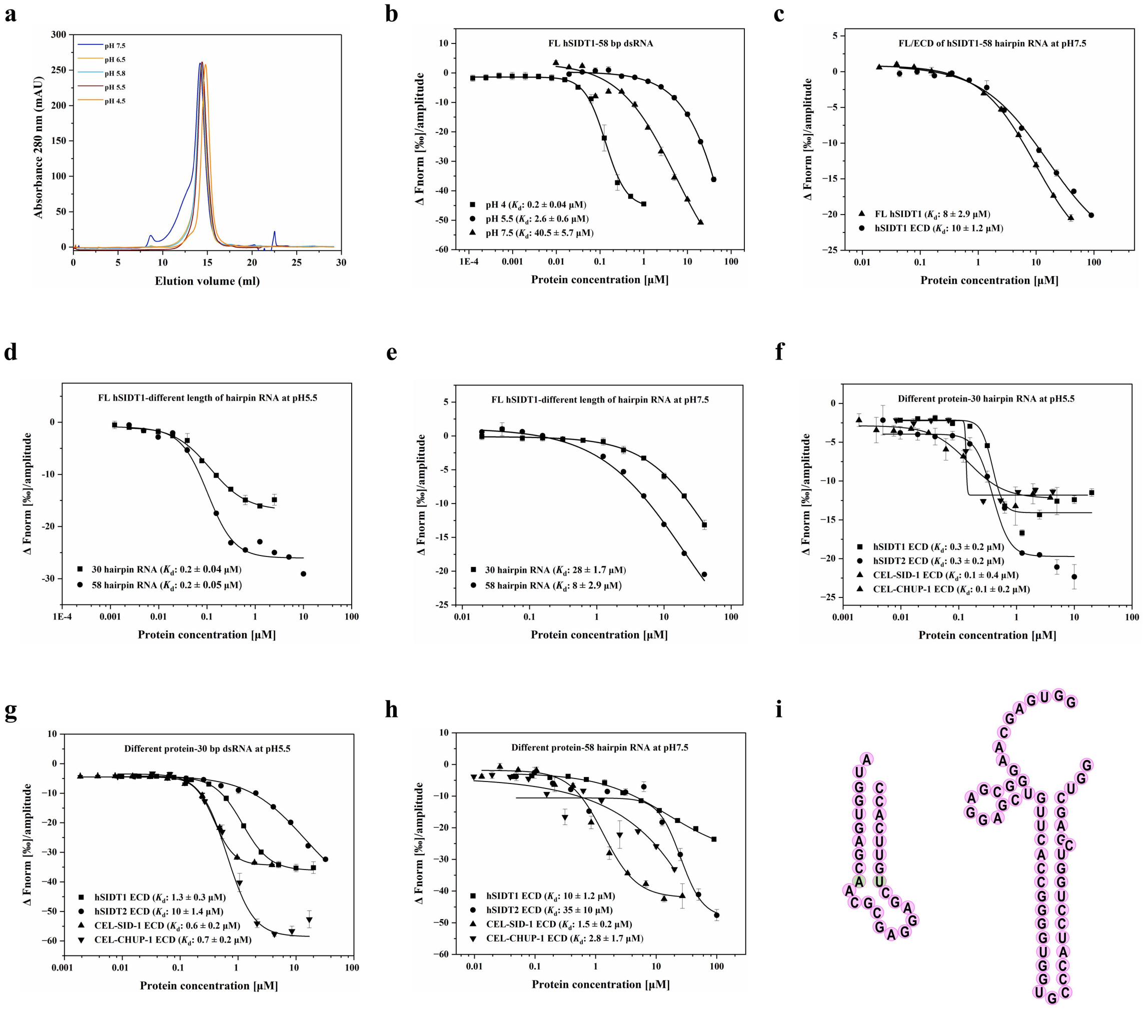

Supplementary Fig. S11 | The binding affinity of hSIDT1 and its homologues with RNA measured by MST. The calculated dissociation constant (K_d) value was shown in the diagram. **a,** Size exclusion chromatography of hSIDT1 under different pH conditions indicated that the stability and homogeneity of hSIDT1 was not affected by pH variation. **b,** pH-dependent binding of hSIDT1 with dsRNA. **c,** The binding affinity of FL hSIDT1 and hSIDT1 ECD with 58 mer hairpin RNA under pH7.5. **d and e,** The binding affinity of hSIDT1 with different length of RNA under pH 5.5 (d) and pH 7.5 (e), respectively. hSIDT1 shows strong binding affinity to RNA of various lengths under pH 5.5, but prefers long-chain RNA at pH 7.5. **f and g,** The binding affinity of hSIDT1, hSIDT2, CEL-SID-1 and CEL-CHUP-1 ECD with 30 mer hairpin RNA (f) and 30 bp dsRNA (g) under pH 5.5.

h, The binding affinity of hSIDT1, hSIDT2, CEL-SID-1 and CEL-CHUP-1 ECD with 58 mer hairpin RNA under pH 7.5. **i,** The secondary structure of 30 and 58 mer hairpin RNA.

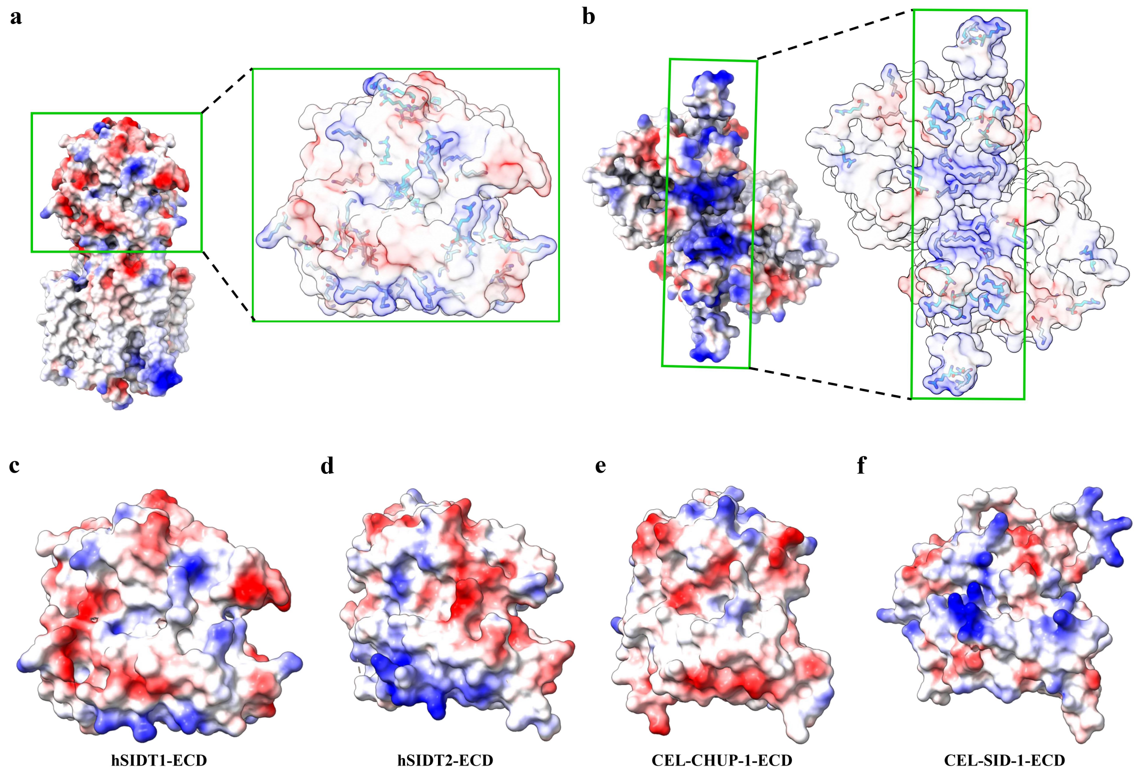

Supplementary Fig. S12 | The electrostatic potential surfaces of hSIDT1 and its homologues. a and b, Two potential RNA binding sites in hSIDT1, which were situated in the extracellular and intracellular regions, were boxed and the positive charged Arg and Lys were depicted as sticks in the transparent surface and colored by cyan and cornflower blue, respectively. **c-f,** The potential RNA binding regions in the ECD of hSIDT1 and its homologues were depicted by electrostatic potential surfaces. The distribution of positively charged residues in the surfaces of ECDs are distinct from each other, suggesting potentially different binding modes for RNAs.

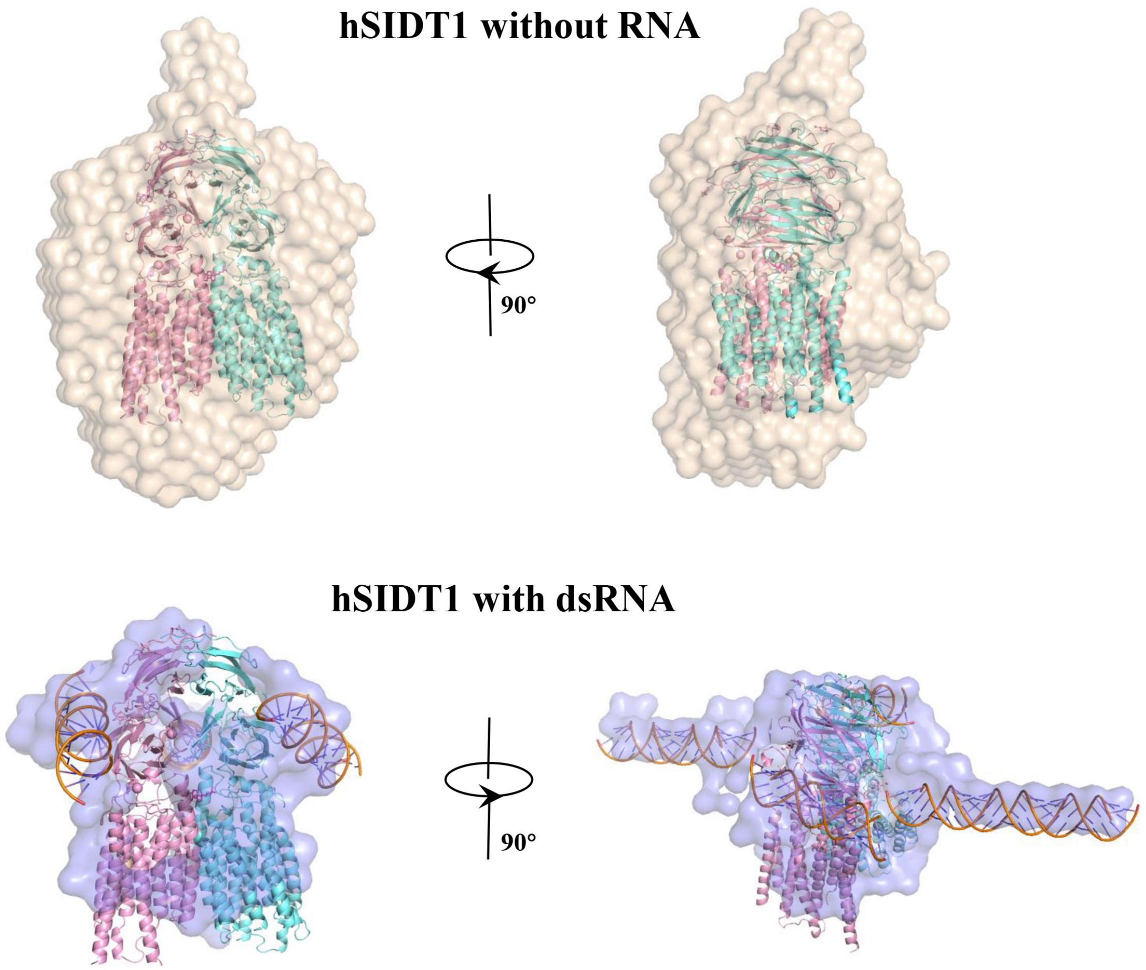

Supplementary Fig. S13 | The binding of hSIDT1 with 45 bp dsRNA by small angle X-ray scattering (SAXS) measurements. a, The 3D model of hSIDT1 in the absence of RNA. **b,** The 3D model of the hSIDT1-dsRNA complex.

a b

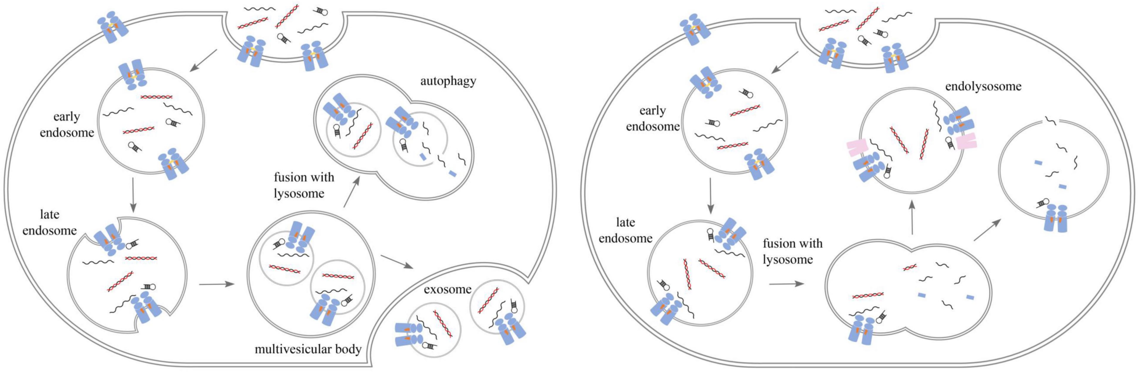

Supplementary Fig. S14 | Potential RNA transport pathways. **a,** hSIDT1 recognizes and captures RNA via its extracellular domain (ECD). The hSIDT1-RNA complex is then internalized into subcellular vesicles through endocytosis. Once internalized, the RNA can be secreted into neighboring cells through exosome. The formation and transport of exosome appear to be influenced by the ceramidase and cholesterol-binding activities of hSIDT1. Alternatively, internalized SIDT1-RNA complex might undergo degradation through the lysosomal autophagy pathway. **b,** The extracellular RNAs are internalized into the intracellular vesicles which fuse with lysosomes subsequently, allowing enhanced protein-RNA interactions in the lysosomal compartments at the low pH. One plausible mechanism is that hSIDT1 may serve as a receptor which delivers RNA into the cytoplasm with assistance of unknown protein partners. Alternatively, hSIDT1 hydrolyses ceramide or other lipid components, inducing the accumulation of hydrolysis products and alterations in the membrane composition, which might affect the integrity of the vesicle membranes. Once the vesicles are collapsed, the pH in the lumen of the vesicles become neutral as in the cytosol, so that the RNAs will be dissociated from hSIDT1 and leak into the cytosol.

Supplementary Table 1 | Cryo-EM data collection, refinement and validation statistics

	hSIDT1$^{pH7.5}$-C1 (EMD-37695, PDB 8WOQ)	hSIDT1$^{pH7.5}$-C2 (EMD-37696, PDB 8WOR)	hSIDT1$^{pH5.5}$-C1 (EMD-37697, PDB 8WOS)	hSIDT1$^{pH5.5}$-C2 (EMD-37698, PDB 8WOT)
**Data collection and processing**				
Magnification	105,000	105,000	105,000	105,000
Voltage (kV)	300	300	300	300
Electron exposure (e–/Å^2)	52.52	52.52	52.52	52.52
Defocus range (μm)	-1.0 to -1.5	-1.0 to -1.5	-1.0 to -1.5	-1.0 to -1.5
Pixel size (Å)	0.851	0.851	0.851	0.851
Symmetry imposed	C1	C2	C1	C2
Initial particle images (no.)	12,083	12,083	13,862	13,862
Final number of particles (no.)	503,286	503,286	146,647	146,647
Map resolution (Å)	2.85	2.66	3.37	3.18
FSC threshold	0.143	0.143	0.143	0.143
**Refinement**				
Model composition				
Nonhydrogen atoms	11,218	11,218	10,672	10,672
Protein residues	1,294	1,294	1,272	1,272
Ligands	35	35	22	22
B factors (Å^2)				
Protein	147.51	111.51	122.93	115.00
Ligand	163.62	129.10	153.69	127.54
R.m.s. deviations				
Bond lengths (Å)	0.003	0.004	0.003	0.003
Bond angles (°)	0.684	0.647	0.716	0.597
Validation				
MolProbity score	1.80	1.78	1.94	1.91
Clashscore	8.59	8.01	11.07	9.52
Poor rotamers (%)	0.43	0.52	0.44	0.35
Ramachandran plot				
Favored (%)	95.21	94.98	94.43	93.87
Allowed (%)	4.79	5.02	5.57	6.13
Disallowed (%)	0.00	0.00	0.00	0.00

References

1 Jamali, K. *et al. BioRxiv*, doi:10.1101/2023.05.16.541002 (2023).

2 Emsley, P. & Cowtan, K. *Acta Crystallogr D Biol Crystallogr* **60**, 2126-2132 (2004).

3 Pettersen, E. F. *et al. J Comput Chem* **25**, 1605-1612 (2004).
4 Adams, P. D. *et al. Acta Crystallogr D* **66**, 213-221 (2010).
5 Schrodinger, LLC. *The PyMOL Molecular Graphics System, Version 1.8* (2015).
6 Svergun, D. I. *J Appl Crystallogr* **25**, 495-503 (1992).
7 Bedia, C., Casas, J., Garcia, V., Levade, T. & Fabrias, G. *Chembiochem* **8**, 642-648 (2007).
8 Petoukhov, M. V. *et al. J Appl Crystallogr* **45**, 342-350 (2012).
9 Franke, D. & Svergun, D. I. *J Appl Crystallogr* **42**, 342-346 (2009).
10 Volkov, V. V. & Svergun, D. I. *J Appl Crystallogr* **36**, 860-864 (2003).
11 Wolfrum, C. *et al. Nat Biotechnol* **25**, 1149-1157 (2007).
12 Mendez-Acevedo, K. M., Valdes, V. J., Asanov, A. & Vaca, L. *Sci Rep* **7**, 7450(2017).
13 Whangbo, J. S., Weisman, A. S., Chae, J. & Hunter, C. P. *G3 (Bethesda)* **7**, 3887-3899 (2017).
